# Community-engaged curriculum development using racial justice and biomedical lenses to address COVID-19 vaccine hesitancy in black individuals with rheumatologic conditions

**DOI:** 10.3389/fpubh.2024.1493331

**Published:** 2025-02-17

**Authors:** Eseosa Olive Osaghae, Greta Sirek, Tonya Roberson, Mia Chandler, Ariel Childs, Monica Crespo-Bosque, Gina Curry, Amar Dhand, Mary Dollear, Alice Eggleston, Nnenna Ezeh, Dieufort Fleurissaint, Denice Garrett, Gail Granville, Muriel Jean-Jacques, Elena Losina, Holly Milaeger, Lutfiyya Muhammad, Mary Ann Nelson, Chisa Nosamiefan, Bisola Ojikutu, Neil Pillai, Mary Beth Son, Marie Jacques Toussaint, Ana Valle, Jessica N. Williams, Michael York, Karen Mancera-Cuevas, Candace H. Feldman, Rosalind Ramsey-Goldman

**Affiliations:** ^1^Division of Rheumatology, Department of Medicine, Northwestern University Feinberg School of Medicine, Chicago, IL, United States; ^2^Division of Rheumatology, Inflammation and Immunity, Department of Medicine, Brigham and Women’s Hospital, Boston, MA, United States; ^3^Governors State University College of Health and Human Services, University Park, IL, United States; ^4^The Rheumatology Program, Boston Children’s Hospital, Boston, MA, United States; ^5^Vital CxNs, Boston, MA, United States; ^6^Department of Rheumatology, Boston Medical Center, Boston, MA, United States; ^7^Office of Community Engagement and Cancer Health Equity, Comprehensive Cancer Center, University of Chicago Pritzker School of Medicine, Chicago, IL, United States; ^8^Division of Neurology, Brigham and Women’s Hospital, Boston, MA, United States; ^9^Lupus Society of Illinois, Chicago, IL, United States; ^10^Alliance Chicago, Chicago, IL, United States; ^11^Department of Internal Medicine and Dermatology, Brigham and Women’s Hospital, Boston, MA, United States; ^12^True Alliance Center, Inc., Boston, MA, United States; ^13^Action for Boston Community Development, Inc., Boston, MA, United States; ^14^Mattapan Community Development Corp, Women of Courage, Boston, MA, United States; ^15^Division of General Internal Medicine, Department of Medicine, Northwestern University Feinberg School of Medicine, Chicago, IL, United States; ^16^Harvard Medical School, Boston, MA, United States; ^17^The Orthopedic and Arthritis Center for Outcomes Research, Department of Orthopedics, Brigham and Women’s Hospital, Boston, MA, United States; ^18^Division of Biostatistics, Department of Preventive Medicine, Northwestern University Feinberg School of Medicine, Chicago, IL, United States; ^19^Mission Hill Health Movement Inc., Roxbury, MA, United States; ^20^The Labalaba Foundation for Lupus Advocacy and Awareness, South Weymouth, MA, United States; ^21^Boston Public Health Commission, Boston, MA, United States; ^22^Division of Global Health Equity, Brigham and Women’s Hospital, Boston, MA, United States; ^23^Division of Rheumatology, Department of Medicine, Emory School of Medicine, Atlanta, GA, United States; ^24^Department of Health Equity, National Health Council, Washington, DC, United States

**Keywords:** community academic partnerships, COVID-19, African American/black, community health promotion, health equity, rheumatic and autoimmune disease, vaccine hesitancy

## Abstract

Despite the efficacy of the COVID-19 vaccine in reducing mortality and illness severity, racial inequities in vaccination uptake persist. Among individuals with rheumatologic conditions who are often immunocompromised, the impact of disparities in preventive care threatens to widen existing inequities in adverse outcomes related to COVID-19 infection. There exists an urgent need to develop interventions that reduce COVID-19 vaccine hesitancy and promote vaccine uptake. We leveraged long-standing community-academic partnerships in two cities to develop a curriculum that will be part of an intervention to decrease COVID-19 vaccine hesitancy within Black communities. We describe the collaborative efforts that resulted in the creation of two interactive virtual curricula with similar core content but different theoretical lenses. One lens uses a racial justice approach to acknowledge the effects of historical and current structural racism on vaccine hesitancy, the other utilizes a traditional biomedical lens. In a future trial, we will compare the efficacy of these curricula to empower Black individuals identified as Popular Opinion Leaders (POLs), or trusted community members with large social networks, to disseminate health information to promote COVID-19 vaccine uptake. Strategies to reduce racial inequities in COVID-19 vaccine uptake must begin with accurately identifying and empathetically acknowledging the root causes of vaccine hesitancy, as well as addressing nuanced concerns that drive vaccine avoidance among Black individuals. Community engagement and collaboration are central in creating interventions to develop and test culturally relevant strategies, as observed with our curricula, that bridge scientific efforts with community concerns and practices.

## Introduction

With an estimated death toll of 14.8 million globally and approximately 1.1 million lives lost along with 6.4 million hospitalizations in the United States alone, the COVID-19 pandemic profoundly impacted the world on an unprecedented scale and specifically revealed many shortcomings present within the United States healthcare system (1, 2). Currently, historically marginalized populations remain disproportionately affected by the COVID-19 pandemic. Accounting for nearly 13% of the United States population, individuals of African ancestry, here after referred to as Black, are more likely to contract COVID-19 and experience adverse long-term outcomes (3–5). With higher hospitalization rates, these individuals are more likely to require intensive care unit admission, mechanical ventilation, and have an 11% higher mortality rate than their white counterparts (3, 6, 7). It is essential to note that there is significant heterogeneity within the Black population and many statistics related to COVID-19 do not specifically address ancestry (5). Yet, despite the efficacy of the COVID-19 vaccine and its subsequent boosters, we continue to see this population have lower COVID-19 vaccination rates and report more hesitancy to get vaccinated or receive a booster (8–10). As one of the top threats to global health as described by the World Health Organization, vaccination hesitancy refers to “*a delay or refusal to accept vaccination despite its availabilit*y (11).” At the height of the pandemic, misinformation and the rise of anti-vaccination movements bolstered an increase in global vaccine hesitancy and avoidance (12). Though U.S.-based Black individuals were not exempt from this phenomenon, the roots of vaccination hesitancy in this group extend past misinformation and harken back to historical instances of unethical and unjust practices in the healthcare system that have ultimately bred mistrust and avoidance (12).

The pervasiveness of vaccination hesitancy within this population is concerning as certain rheumatic conditions like systemic lupus erythematosus (SLE) disproportionately affect Black individuals, and require immunosuppressive therapies, which heighten risk of severe infection (13, 14). Studies have demonstrated reduced efficacy of the COVID-19 vaccine in immunosuppressed individuals, highlighting the importance both of booster vaccinations and of advocacy to vaccinate not only individuals with rheumatic conditions but also their close contacts (15). Thus, our future intervention trial focuses on addressing and ultimately, decreasing vaccine hesitancy among Black individuals with rheumatic conditions.

**Figure 1:**
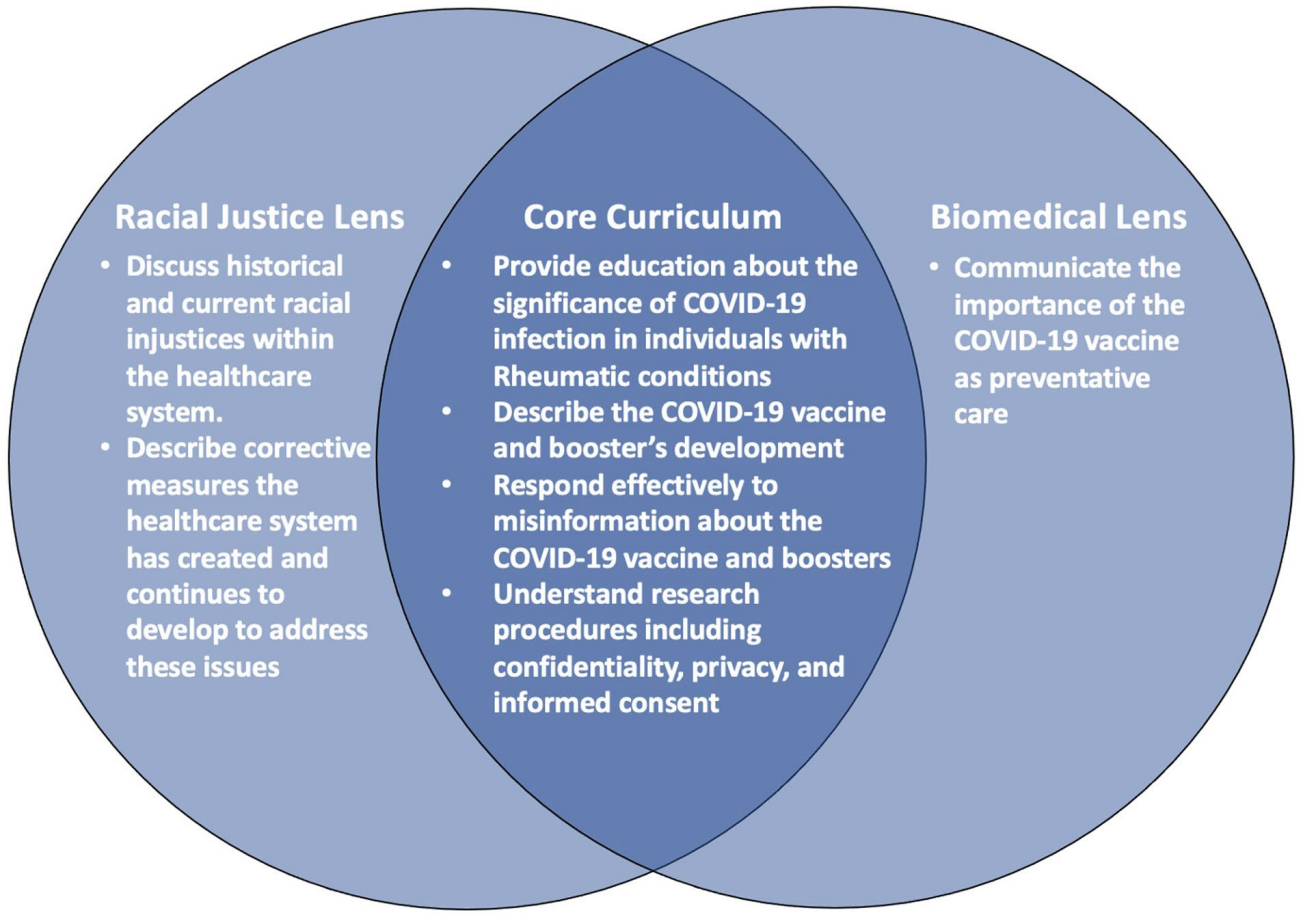
Objectives of two virtual curricula embedded with different theoretical frameworks.

In this paper, we describe the process of developing two virtual curricula, informed by the racial justice and biomedical models, respectively, with collaboration between longstanding academic and community partners in two cities. These curricula contain similar core content with two different lenses and objectives (Figure 1; Table 1). We ultimately aim to use these curricula to train Popular Opinion Leaders (POLs), or trusted community leaders, to disseminate health information to their social network members. Our forthcoming trial utilizes the POL model, an evidence-based and community-based approach previously used to reduce HIV stigma and increase SLE awareness (16, 17). Grounded in the social network and diffusion of innovation theories, the POL model trains community leaders to engage their social network members in health-related discussions that ultimately lead to adoption of positive health norms and behaviors (17, 18). Thus, our primary goals are to influence the content of these discussions through developing curricular material that trains POLs while determining the efficacy of two distinct curricular perspectives - biomedical versus racial justice – in training these POLs to effectively disseminate information concerning the COVID-19 vaccine. This dissemination is intended to decrease COVID-19 vaccine hesitancy and increase vaccine uptake among their social network members. This paper’s objective is to illustrate our community-engaged, iterative approach to design these two curricula and their relevant pre- and post-tests for use in this planned NIH-funded randomized clinical trial (Northwestern University Institutional Review Board (IRB, ID #STU00217038) and Mass General Brigham IRB (#2022P000633 and #2023P000686) (19).

**Table 1:** Summary of each module.

Racial justice lens	Biomedical lens
Module I: Introduction & background	
POL model (what it is, how to influence community, how to communicate, how to measure success, etc.)	
Framed with emphasis on racial justice to reduce mistrust, add transparency to the vaccination process, change cultural norms and behaviors to reduce inequities in vaccine uptake at the population level	Framed with role of health information/education dissemination to change individual beliefs & behaviors around vaccine uptake
Module II: COVID-19 infection risk & vaccine mechanisms	
Part A: Infection risk in rheumatic diseases, highlighting racial & socioeconomic inequities in COVID-19 infections and vaccine use; discussions of other infections (e.g., influenza) and vaccines Part B: COVID vaccine mechanisms, safety, efficacy, and side effect data with trial data from racial/ethnic minority individuals	Part A: Infection risk in rheumatic diseases; discussions of other infections (e.g., influenza) and vaccines Part B: COVID vaccine mechanisms, safety, efficacy, and side effect data with trial data
Module III: Vaccine-related myths and evidence-based responses	
Common myths include beliefs regarding the speed of COVID-19 vaccine development and the likelihood or possibility of developing severe side effects such as stroke, infertility, stroke, and sudden death after receiving the COVID-19 vaccine. Other myths involve the ingredients within the COVID-19 vaccine being unsafe and concerns that the COVID-19 vaccine is a government ploy.	
Addresses concerns regarding racial diversity in COVID-19 vaccine clinical research	Addresses commonly held myths regarding the COVID-19 generally
Module IV: Structural racism, racial inequities in infection risk and preventative care uptake	Module IV: General preventative care
Part A: Discrimination in healthcare Part B: Historical injustices in medical research, and protection in place for patients	Part A: Discuss Unhealthy health practices Part B: Examples of preventative care
Module V: Research methods	
Human Subjects Training, Collecting Research Data, Data collection procedures, HIPAA, confidentiality	
Module VI: Review of material role-play with common questions	
General conversation strategies (who, what, when, where) Examples: storytelling, direct language, iterative conversations Role play including consenting and dissemination of information	
Racial justice-framed conversation starters	Conversation starters incorporating preventative care strategies

## Theoretical framework

### Curricula strategy: biomedical model or racial justice model

To decrease COVID-19 vaccine hesitancy, current strategies commonly emphasize educating individuals about COVID-19 through development of innovative training materials. These teaching materials are often framed with a biomedical lens highlighting the vaccine as an individual-level tool to prevent serious infection. This model proposes that illness primarily arises as an outcome of abnormal biology or deviations from normal physiological function with psychological or sociological factors having less significant roles (20). With the biomedical approach, the COVID-19 vaccine is placed among other trusted preventative health practices with vaccine hesitancy addressed primarily by acknowledging and addressing scientific concerns. An example of a strategy utilizing the biomedical model involved the creation of a digital intervention through individualized motivational interviewing techniques that addressed COVID-19 vaccine misinformation and provided education about its development following a systematic literature review and qualitative interviews with public health experts (21).

Previously published work that involved semi-structured interviews with physicians and community leaders to determine barriers toward COVID-19 vaccination for Black individuals identified strategies that differed from the biomedical model to decrease COVID-19 vaccine hesitancy (22). These strategies emphasized the importance of acknowledging racial, ethnic and socioeconomic injustices, using compassionate and motivational messaging, and addressing misinformation, that is, taking a racial justice-oriented approach (22). Unlike the biomedical approach, the racial justice model recognizes and openly acknowledges the role of current and historical racial and social inequities in poor health outcomes, focusing on population-level motivations and goals as opposed to individual-level objectives (23). This model contends that acknowledging and addressing the psychological and sociological health of Black communities is an essential factor to reduce health inequities. We see this strategy utilized by Peteet et al. (24) with the development of a webinar for Black churchgoers that discussed the psychology behind the fear of the COVID-19 vaccine by acknowledging medical mistrust. Another study reported the development of a vaccine education campaign focused on transparency and having culturally sensitive discussions regarding COVID-19 vaccine hesitancy with Black employees of nursing homes and their social networks (25). Though both interventions successfully decreased COVID-19 vaccine hesitancy in their respective populations, neither study compared racial justice-focused strategies to the norm, a biomedical-based educational approach to understand if there was a difference in efficacy between these two models. Therefore, to develop innovative strategies to reduce COVID-19 vaccine hesitancy among Black individuals, it is necessary to determine which educational model is more successful in changing health attitudes and behaviors.

Diffusion of innovation theory, the foundation of the POL model, focuses on the process of how and why innovative ideas and behaviors are adopted in a population (18). It proposes that searching for and creating new methods that better fit existing ideals and needs of hesitant individuals is necessary for behavioral change (18). This theory describes early adopters as individuals who embrace change and cautiously adopt new behaviors, and early and late majorities as individuals who are hesitant, and require more information and time for deliberation (18). Thus, when considering our approach to decrease COVID-19 vaccine hesitancy among Black individuals, we aimed to develop educational materials that were informative and both culturally relevant and sensitive such that Black individuals or POLs who are “early adopters” of the COVID-19 vaccine can influence members of their communities and social networks who might then become part of the “early and late majorities” (18).

## Learning environment

### Curricula audience

These curricula were created to be delivered to Black individuals who are older than 18 years, have a rheumatic and musculoskeletal condition, speak English, and have received at least one COVID-19 vaccine. These individuals will receive curricula training virtually over Zoom meetings.

### Curricula development overview

To develop our curricula, we utilized longstanding collaborations between our research team and academic and community partners from Boston and Chicago. This group consisted of racially and ethnically diverse academic clinicians and researchers, rheumatic disease, infectious disease, and general medicine healthcare providers, neighborhood organization and community leaders, advocacy groups, social workers, and experts in public health. POLs from our previous studies were also significant members in this group. These individuals met monthly through Zoom meetings within and across both cities since summer 2022 and continue to meet.

### Curricula objectives

To create our curricula, we leveraged findings from previous work where a series of semi-structured interviews with physician and community stakeholders focused on finding strategies to address COVID-19 vaccine hesitancy among Black individuals with rheumatic condition (22). Combining information from this work with the lived experiences and expertise of our community and academic partners, we planned to develop curricula that would accomplish the objectives as seen in Figure 1 (22).

Following the development of the learning objectives, we used multiple methods to review existing literature to understand inequities in vaccine uptake and adverse COVID-19 outcomes, as well as historical racial injustices. Meeting with our community and academic partners to discuss the concerns and questions they had heard about the COVID-19 vaccine from their patients and community members informed further literature review and our curricula drafts.

**Figure 2:**
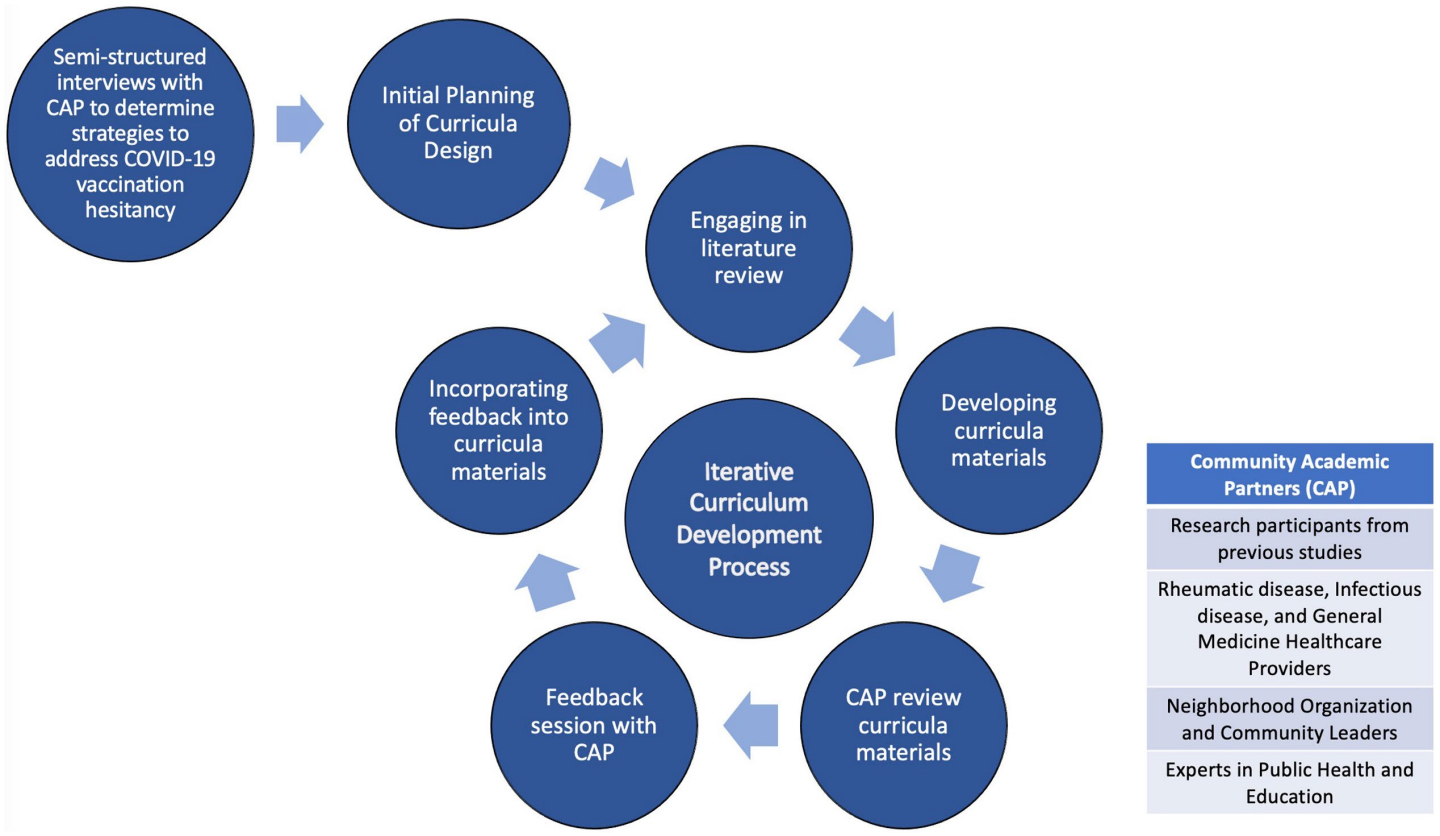
Iterative process of curricula development.

After creating the curricula’s initial drafts, developed using Microsoft PowerPoint, we sent the materials to our community and academic partners for review. Each partner also received a worksheet that allowed them to reflect and comment on their thoughts and concerns after reviewing the slides. This worksheet contained guided questions such as, “please list any slides you found confusing,” “suggest how the slides can be improved,” and “were the goals of this module clear?” After the partners completed their review, our research team met with them over Zoom meetings to discuss any suggestions and feedback. This process was repeated numerous times during the development of our curricula to ensure the curricula met our objectives. Throughout this iterative process to develop educational materials that accurately accomplished our learning objectives, the primary challenges we repeatedly encountered were addressing the curricula’s content, accessibility, and tone (Figure 2).

### Curricula content

Developing both curricula content involved multiple methods to broaden our understanding of COVID-19 incidence and prevalence, vaccine development, vaccination recommendations, and common misconceptions regarding vaccination. Various sources were reviewed and utilized including data from the Centers for Disease Control and Prevention (CDC), articles from the lay press, and peer-reviewed published manuscripts in PubMed indexed journals. Creating the material for the curriculum with a racial justice lens required extensive research about data on bias and mistrust in medicine and historical and current treatment of Black individuals in the health care system and general society. Furthermore, upon receiving feedback from some of our partners regarding tailoring the curriculum content specifically to the sites where we will ultimately deliver the curriculum, we searched for distinct examples of how the healthcare systems in both cities addressed mistrust and medical bias. Additional content to inform the biomedical lens involved review of health screening and preventative guidelines for people with rheumatic conditions (15). Some of our public health experts provided several examples of teaching materials focused on preventative care which were incorporated into our curriculum.

Given the collaborative nature of the curricular development, at times, our community and academic partners had differing perspectives on the curricula’s content. To address these conflicts, we integrated the varying ideas into our curricula drafts and, as part of the iterative process, invited our partners to evaluate which approach best aligned with our overall goals.

### Curricula accessibility

**Figure 3:**
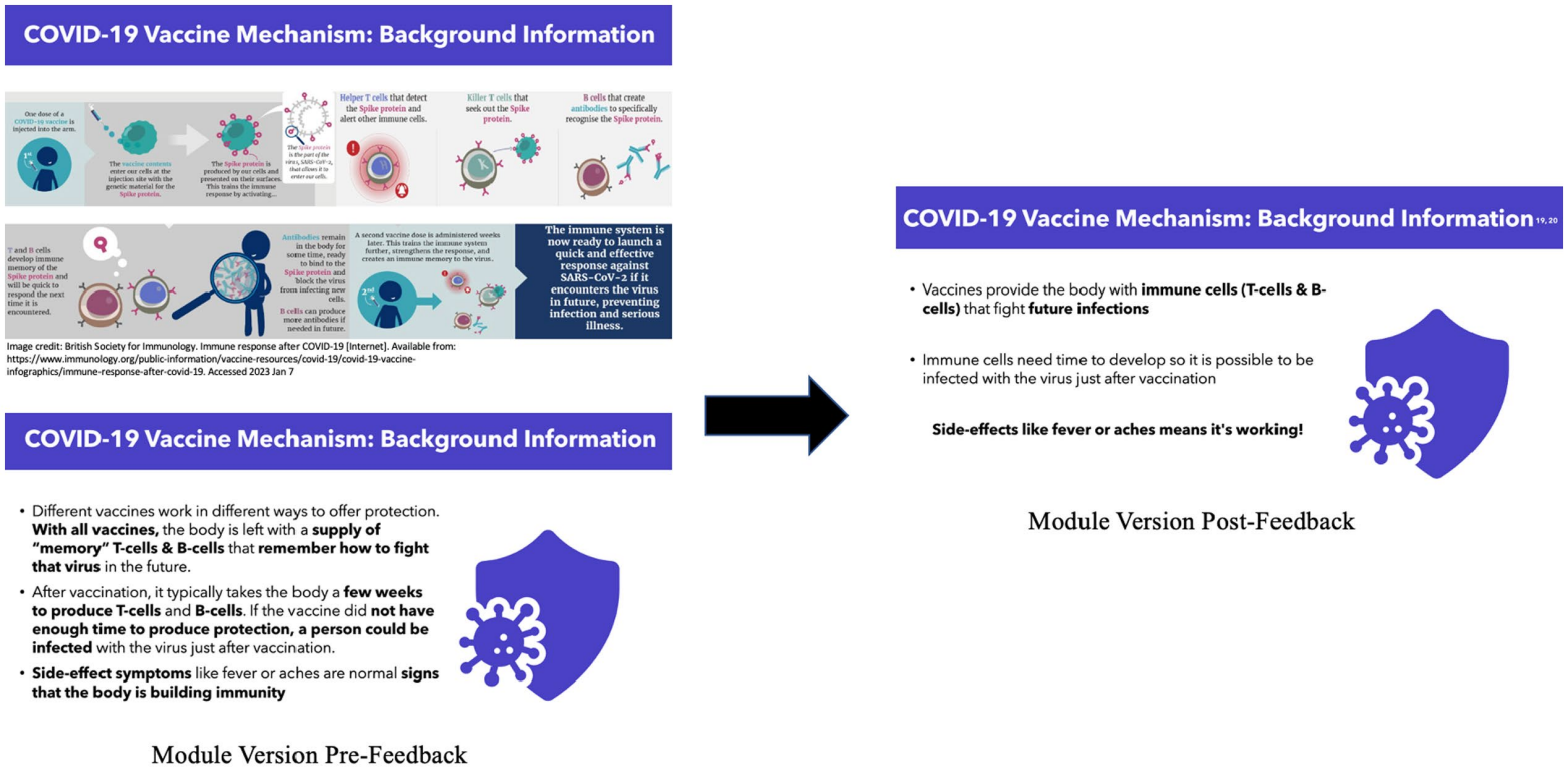
Curricula outcome of addressing feedback regarding literacy level.

During meetings with our community and academic partners, we identified certain language used in our curricula drafts that was too scientific or contained medical jargon, which would limit accessibility of our curricula to a broad audience (Figure 3). To enhance the materials’ comprehensibility and create an approachable learning environment, we revised the language used, replacing technical terminology with understandable terms (Figure 3). Several strategies were used to check on the literacy of the materials including having the curricula reviewed by individuals blinded to the curricula or intervention’s goals. A literacy check was conducted to ensure the language met the desired comprehension level of an 8th-grade student (26). Based on feedback and comments about challenges understanding biomedical terminology, we also created a glossary to define terms such as “beneficence” or “variant” to further achieve this goal.

### Curricula tone

**Figure 4:**
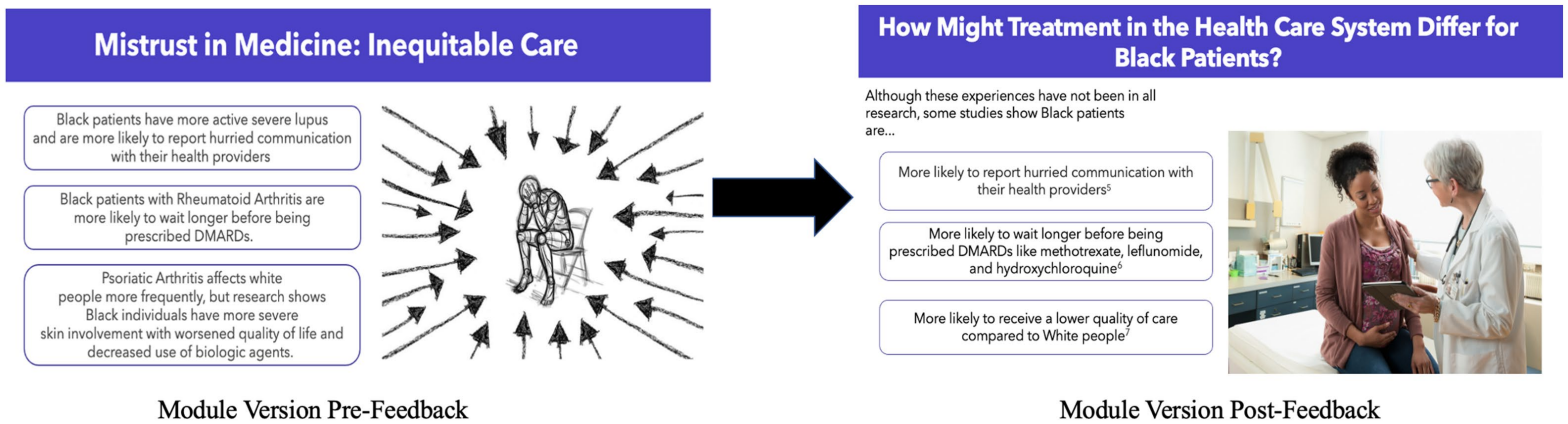
Curricula outcome of addressing feedback about tone. Image 1 is reprinted with permission from “Stress Burnout Despair” by Gerd Altman, licensed under Content License. Image 2 is reprinted with permission from “Gynecologist with digital tablet comforting pregnant patient” by Jose Luis Pelaez under a Royalty-free license.

In our meetings, we frequently discussed the tone of the curricula, particularly the version with the racial justice lens. Initially, the feedback from our community and academic partners was that this curriculum lens could be perceived as disheartening and demoralizing notably when discussing racial bias or discrimination Black individuals encounter. In response, we made this curriculum’s language empowering and removed images that elicited feelings of disillusionment identified by community members as problematic (Figure 4). We changed the language used in the curriculum from having an individualized perspective, removing any individualized culpability and instead, created a sense of collective responsibility. For example, rather than stating, “here’s what you need to do to become healthier,” we revised the curriculum to describe a collective “we” as in “here’s how we can be healthier.” In addition, we added information about current initiatives within the healthcare system that address issues such as medical mistrust and bias to further decrease feelings of frustration or defeat for curricula learners.

We addressed feedback about the lecture-based tone of both curricula by making the modules more interactive with content checks and multiple choice/true or false questions, and specifically created a module to practice anticipated conversations using role play.

## Learning assessment

To assess POLs curricular knowledge retention following training, we followed the format of previous research where a curriculum was developed for POLs to promote familiarity of clinical trials and research methods to Black individuals with Lupus (27). Similar to that study, each module in our curricula contained pre- and post-test questionnaires to assess POLs’ knowledge acquisition. Each curriculum had similar questions testing core competencies, but also contained additional questions specific to that curriculum’s lens. These test questions had a format of multiple choice, short answer, and true or false per feedback from the previous POL study suggesting only utilizing a multiple-choice format was intimidating (27). These questions, along with the curricula slides, were sent to our community and academic partners to review. They were asked to assess the clarity and simplicity of the questions with space to offer suggestions for improvement, which were then incorporated into the pre- and post-tests (Supplementary Table 1).

## Results: curricula design

Following an iterative process with our community and academic partners over a period of a year, we developed two virtual curricula with shared core content, but two different theoretical lenses. Each curriculum contained five distinct modules as described below. The curricula both provide education about the development of the COVID-19 vaccine and address COVID-19 misinformation. The modules with a racial justice lens describe the origins of vaccine hesitancy among the Black population and the health care system’s role informing this belief, whereas the curriculum with a biomedical lens explores the importance of preventative care and highlights the COVID-19 vaccine as a tool in reducing an individual’s adverse health outcomes.

### Modules description

Module I: We described the role of POLs as trusted individuals within their social networks. These modules discussed how these individuals will influence and promote healthy behaviors in their communities.

Module II: We discussed the mechanism and development of the COVID-19 vaccine, detailing its side effects and safety profile (Figure 3). These modules included information about COVID-19 risks, current epidemiology data, and education regarding various rheumatic disease presentations. The curriculum with the racial justice lens differed from the biomedical lens by specifically detailing COVID-19 risk in Black populations and provided trial data about Black individuals’ participation in the development of the COVID-19 vaccine.

Module III: We described common myths and provided evidence-based discussions to dispel misinformation about the COVID-19 vaccine. Some myths discussed were concerns that the COVID-19 vaccine could cause COVID-19 infection and beliefs that there was an increased possibility or likelihood of getting severe side effects from the COVID-19 vaccine such as stroke, infertility, and sudden death. Other myths addressed included concerns about how quickly the COVID-19 vaccine was developed, beliefs about the presence of dangerous ingredients within the vaccine, and notions that the vaccine was a government ploy. The curriculum framed with the racial justice lens also addressed concerns regarding the diversity of individuals involved in developing and participating in the COVID-19 research clinical trials.

Module IV: The racial justice framed curriculum defined structural racism, discussed etiologies of current inequities in the COVID-19 infection risk and severity for the Black population, and addressed healthcare mistrust by acknowledging past injustices within the health care system and current methods for preventing recurrence (Figure 4). Short video recordings and visual art played prominent roles in this module to explain difficult and emotionally charged concepts. The module from the curriculum framed with the biomedical lens discussed general preventative care strategies including highlighting the importance of nutrition, physical activity, cancer screenings, and vaccinations for individuals with rheumatic conditions. The objective of this module was to describe the COVID-19 vaccine as a form of preventative care.

Module V: We emphasized the role POLs play as community researchers. We discussed data collection procedures, highlighting HIPAA and confidentiality.

Module VI: We provided a summary of the prior modules, revisiting any key information previously discussed. These modules included role play to reinforce previous teaching and allow POLs to practice having difficult conversations.

As of December 2024, we have recruited our POLs at both cities and randomized them to either receive the racial justice or biomedical framed curriculum for their training (19). We will begin teaching the POLs virtually mid- December and pre and post-tests will be administered for each module to ensure that the teaching is effective. POLs will then be asked to disseminate the information they learned through their social networks and COVID-19 vaccine uptake among network members will be assessed.

## Discussion: implications for practice

Inequities in COVID-19 vaccination uptake have often been attributed to poorer access to the vaccine and misinformation regarding vaccine safety and testing (28). However, a critical factor is the strained relationship between the United States’ healthcare system and Black communities due to longstanding discrimination and societal and healthcare injustices. Prior studies document that Black individuals are more likely to report hurried communication with their health providers and feel less cared for or listened to by their physicians (29, 30). These experiences coupled with historical and contemporary examples of racism and mistreatment have led to medical mistrust which ultimately play a key role in COVID-19 vaccine hesitancy (31). To address COVID-19 vaccine hesitancy and improve vaccine uptake among Black individuals, we have developed two virtual curricula that will equip community leaders across two cities who identify as Black to disseminate information about the COVID-19 vaccine and acknowledge concerns about the vaccine to hesitant Black individuals given prior and current racial injustices. The objectives of these curricula are to acknowledge the historical and current racial discrimination Black individuals encounter in the healthcare system, address the role these interactions have on COVID-19 vaccine hesitancy, and ultimately empower Black individuals to teach members of their social networks, through transparent and informative discussions, about the importance of getting vaccinated.

One of the innovative aspects of creating these curricula is the utilization of community and academic partnerships at every step of the developmental process to shape the curricula’s content, accessibility, and tone. From previous work involving focus groups with community leaders and physician partners, we know that strategies to improve COVID-19 vaccine uptake in Black populations with rheumatic conditions must be motivational while acknowledging and addressing racial and social injustices (22). Prior studies have described the need for a social justice framework in public health education that adequately recognizes the effects of social inequities on health outcomes (23). Many researchers have developed anti-racist curricula to educate health professionals and equip them with tools to address health inequities (32–34). With our curricula, created by a diverse group of individuals motivated by the common mission to reduce inequities, we step outside of the health system into the community and focus attention on the individuals affected by these inequities to create materials that are directly informed by their perspectives and experiences.

Another benefit of community engaged collaboration is the creative environment these interactions form that leads to the development and implementation of innovative ideas. Currently, the literature is lacking data on which curricular approach, the traditional biomedical or the racial justice model, is more successful for improving COVID-19 vaccine uptake among Black individuals. Creating two adjacent curricula with these two lenses addresses this gap by allowing for direct comparison to determine which perspective leads to knowledge acquisition and which leads to more effective knowledge dissemination to reduce inequities in COVID-19 vaccination. Independent of curricula perspective, developing these curricula addresses the urgent need for researchers in public health to find strategies that reduce COVID-19 vaccination inequities for Black individuals. However, creating a racial justice curriculum specifically acknowledges the population that lives with these inequities and establishes materials that directly caters to this group on a sociological, psychological, and cultural level. The COVID-19 pandemic only highlighted known racial and ethnic health inequities that are deeply rooted in our society. The iterative process used to develop these curricula acknowledges and addresses these inequities while providing useful information for future curricula development and interventions that address other health issues where we find similar inequities.

As with any curricula development, there were notable challenges in this process. One such challenge involved resolving conflicting ideas and suggestions between team members which is inevitable within collaborative efforts. Being cognizant of the etiologies of these suggestions along with the role life experiences played in each person’s perspective led to learning opportunities that enriched this work and future perspectives. Though our curricula discuss concerns Black individuals have about the COVID-19 vaccine, we recognize the great heterogeneity within the United States Black population and note that our curricula do not represent the entirety of diverse perspectives and concerns within this group (5). However, due to structural racism, there are commonalities regarding concerns about the COVID-19 vaccine and the health care system generally that stem from similar experiences of racism that we hope to acknowledge and represent. Moreover, for our future intervention, we plan to recruit a diverse group of POLs to capture aspects of the diversity within the Black population.

Addressing COVID-19 vaccination uptake and decreasing vaccine hesitancy among Black individuals is essential. Strategies to improve health outcomes for Black individuals are most successful when trusted members from their communities are included in the development of these interventions. This collaboration, which can be replicated in other cities and countries, lays the groundwork for transparency and trust which increases the likelihood of creating impactful, effective, and culturally sensitive work.

## Data Availability

The original contributions presented in the study are included in the article/Supplementary material, further inquiries can be directed to the corresponding author.

## Supplementary material

The Supplementary material for this article can befound online at: https://www.frontiersin.org/articles/10.3389/fpubh.2024.1493331/full#supplementary-material
