# Corrigendum: Community-engaged curriculum development using racial justice and biomedical lenses to address COVID-19 vaccine hesitancy in black individuals with rheumatologic conditions

**DOI:** 10.3389/fpubh.2025.1638460

**Published:** 2025-06-30

**Authors:** Eseosa Olive Osaghae, Greta Sirek, Tonya Roberson, Mia Chandler, Ariel Childs, Monica Crespo-Bosque, Gina Curry, Amar Dhand, Mary Dollear, Alice Eggleston, Nnenna Ezeh, Dieufort Fleurissaint, Denice Garrett, Gail Granville, Muriel Jean-Jacques, Elena Losina, Holly Milaeger, Lutfiyya Muhammad, Mary Ann Nelson, Chisa Nosamiefan, Bisola Ojikutu, Neil Pillai, Mary Beth Son, Marie Jacques Toussaint, Ana Valle, Jessica N. Williams, Michael York, Karen Mancera-Cuevas, Candace H. Feldman, Rosalind Ramsey-Goldman

**Affiliations:** ^1^Division of Rheumatology, Department of Medicine, Northwestern University Feinberg School of Medicine, Chicago, IL, United States; ^2^Division of Rheumatology, Inflammation and Immunity, Department of Medicine, Brigham and Women's Hospital, Boston, MA, United States; ^3^Governors State University College of Health and Human Services, University Park, IL, United States; ^4^The Rheumatology Program, Boston Children's Hospital, Boston, MA, United States; ^5^Vital CxNs, Boston, MA, United States; ^6^Department of Rheumatology, Boston Medical Center, Boston, MA, United States; ^7^Office of Community Engagement and Cancer Health Equity, Comprehensive Cancer Center, University of Chicago Pritzker School of Medicine, Chicago, IL, United States; ^8^Division of Neurology, Brigham and Women's Hospital, Boston, MA, United States; ^9^Lupus Society of Illinois, Chicago, IL, United States; ^10^Alliance Chicago, Chicago, IL, United States; ^11^Department of Internal Medicine and Dermatology, Brigham and Women's Hospital, Boston, MA, United States; ^12^True Alliance Center, Inc., Boston, MA, United States; ^13^Action for Boston Community Development, Inc., Boston, MA, United States; ^14^Mattapan Community Development Corp, Women of Courage, Boston, MA, United States; ^15^Division of General Internal Medicine, Department of Medicine, Northwestern University Feinberg School of Medicine, Chicago, IL, United States; ^16^Harvard Medical School, Boston, MA, United States; ^17^The Orthopedic and Arthritis Center for Outcomes Research, Department of Orthopedics, Brigham and Women's Hospital, Boston, MA, United States; ^18^Division of Biostatistics, Department of Preventive Medicine, Northwestern University Feinberg School of Medicine, Chicago, IL, United States; ^19^Mission Hill Health Movement Inc., Roxbury, MA, United States; ^20^The Labalaba Foundation for Lupus Advocacy and Awareness, South Weymouth, MA, United States; ^21^Boston Public Health Commission, Boston, MA, United States; ^22^Division of Global Health Equity, Brigham and Women's Hospital, Boston, MA, United States; ^23^Division of Rheumatology, Department of Medicine, Emory School of Medicine, Atlanta, GA, United States; ^24^Department of Health Equity, National Health Council, Washington, DC, United States

**Keywords:** community academic partnerships, COVID-19, African American/black, community health promotion, health equity, rheumatic and autoimmune disease, vaccine hesitancy

In the published article, an author name was incorrectly written as Alice Eggelston. The correct spelling is Alice Eggleston. The correct author list should be written as:

Eseosa Olive Osaghae, Greta Sirek, Tonya Roberson, Mia Chandler, Ariel Childs, Monica Crespo-Bosque, Gina Curry, Amar Dhand, Mary Dollear, Alice Eggleston, Nnenna Ezeh, Dieufort Fleurissaint, Denice Garrett, Gail Granville, Muriel Jean-Jacques, Elena Losina, Holly Milaeger, Lutfiyya Muhammad, Mary Ann Nelson, Chisa Nosamiefan, Bisola Ojikutu, Neil Pillai, Mary Beth Son, Marie Jacques Toussaint, Ana Valle, Jessica N. Williams, Michael York, Karen Mancera-Cuevas, Candace H. Feldman, Rosalind Ramsey-Goldman

The original article has been updated.

