# Supplementary material for: Community-engaged curriculum development using racial justice and biomedical lenses to address COVID-19 vaccine hesitancy in black individuals with rheumatologic conditions
Source: Front Public Health. 2025 Feb 17;12:1493331. doi: 10.3389/fpubh.2024.1493331 (PMC11874835; doi:10.3389/fpubh.2024.1493331)
Supplement: Supplementary file 1 [file Data_Sheet_1.PDF]

Table 1:

Examples of Pre/Post Modules Questionnaires

<table>
<tr><th>RACIAL JUSTICE LENS</th><th>BIOMEDICAL LENS</th></tr>
<tr><td colspan="2">Module I: Introduction & Background</td></tr>
<tr><td colspan="2">Which of the following describes the ideal background and qualities that are needed to be a popular opinion leader?
○ A person who has a medical degree.
○ A respected, well-informed, trusted member of a community
○ A person who has a lot of friends

Which of the following are ways you as a Popular Opinion Leader can measure success in your community and social networks? Please choose all that apply.
○ Write down conversations I had with people
○ Take notes during meetings with organizations
○ Complete study surveys

Please describe a few people or groups within your social network.</td></tr>
<tr><td colspan="2">Module II: COVID-19 Infection Risk & Vaccine Mechanisms</td></tr>
<tr><td colspan="2">The COVID-19 was developed rapidly in comparison to other vaccines. Please list a few ways the COVID-19 was safely expedited.

Vaccines work by:
○ Producing “memory” T cells & B cells that remember how to fight infection
○ Altering our DNA</td></tr>
<tr><td colspan="2">Modules III: Vaccine-related Myths and Evidence-based Responses</td></tr>
<tr><td colspan="2">A community member approaches you concerned that “the COVID-19 vaccine will give them COVID-19.” How might you address this concern with them?

The COVID-19 vaccine has been shown to affect fertility.
○ True
○ False</td></tr>
<tr><td>Module IV: Structural Racism, Racial Inequities in Infection Risk and Preventative Care Uptake</td><td>Module IV: General Preventative Care</td></tr>
<tr><td>A community member states she is not getting the COVID-19 vaccine because medicine, as a whole, is racist. What would you say in response?

In a few words, how would you define structural racism?</td><td>Which of the following actions can improve one’s overall health?
○ Increased physical activity
○ Getting vaccinated
○ Getting routine cancer screenings
○ All of the above

In a few words, what foods make up a healthy diet?</td></tr>
<tr><td colspan="2">Modules V: Research Methods</td></tr>
<tr><td colspan="2">True or false, the institutional review board only reviews research studies if a participant complains or becomes sick?

In a sentence, please describe how you will get informed consent from your social network member.

True or false: If someone agrees to participate in this study but then changes their mind and decides they don’t want to continue, they are obligated to continue the study.</td></tr>
</table>
